# Causes of early hospital readmission in hospice patients at a comprehensive oncology center in Jordan: a retrospective descriptive study

**DOI:** 10.1186/s12904-026-02075-3

**Published:** 2026-04-01

**Authors:** Ala’a Dabbous, Areen Said, Amani El Mughrabi, Fadi Abu Farsakh, Sewar S Salmany

**Affiliations:** 1 Department of Pharmacy, King Hussein Cancer Center, Amman, Jordan; 2 Department of Palliative Care, King Hussein Cancer Center, Amman, Jordan

**Keywords:** Hospice, Palliative Care, Hospital readmission, Jordan, End-of-life care, Oncology

## Abstract

**Background:**

Early readmissions among hospice patients remain common, despite goals to minimize them. This issue remains underexplored in the Middle East. This study describes the primary causes of hospital readmission from hospice settings and reports patient characteristics observed among readmitted cases at a comprehensive oncology center in Jordan.

**Results:**

A total of 163 readmissions were analyzed, representing 133 unique patients. The median age was 59 years (range: 18–88), and 51.9% were female. 72.2% had at least one comorbidity, predominantly cardiovascular (83.3%). Gastrointestinal cancers were the most common primary diagnosis (27.8%). Prior to readmission, 71.8% were receiving home hospice care, and 63.8% had Full Code status. The median Palliative Performance Scale was 40. Uncontrolled conditions accounted for 56.4% of readmissions, primarily worsening infections, and uncontrolled pain, while unanticipated new medical issues contributed to 52.8%. Caregiver distress was documented in 12.3% of cases. More than half of patients (54.0%) died during hospitalization.

**Conclusions:**

Within seven days of discharge, hospice patients in our center were readmitted most commonly for uncontrolled symptoms and new medical issues. These descriptive findings may help target quality improvement in symptom management, discharge planning, documentation of end‑of‑life preferences, and transitions between hospice and hospital services.

**Trial registration:**

Not applicable.

## Background

Palliative care can be provided alongside disease‑directed therapy at any stage of serious illness. Hospice care is a model of care focusing on comfort and quality of life for patients with limited life expectancy, typically when the goals of care prioritize symptom relief rather than disease‑directed treatment. Hospice is an approach to care that is not tied to a specific place and can be delivered at home, in hospitals, nursing homes, or dedicated facilities. At our institution, we provide different settings of hospice and palliative care (HPC) services, including inpatient wards, outpatient clinics, and home care services.

Hospice care is delivered by a multidisciplinary team with specific skills, including physicians, nurses, social workers, spiritual advisors, and clinical pharmacists. Everyone collaborates to provide medical, emotional, and spiritual support to patients and families. A member of the hospice team visits regularly, and the team is always available over the phone — 24 h a day, seven days a week [1].

Avoiding hospitalization typically aligns with hospice philosophy and many patients’ preferences to remain at home [2, 3]. Nevertheless, despite these preferences, hospital readmissions still occur within a short interval after discharge [4].

Readmissions pose a significant burden to patients and healthcare systems due to increased mortality, morbidity and healthcare costs. The latter is not only important regarding well-being but also has an important economic impact in the form of costly hospitalization [5]. Globally, few studies have evaluated reasons for readmission and characteristics of patients readmitted from hospice. To our knowledge, neither was assessed in the Middle East, where service is still developing and has not been thoroughly evaluated.

Palliative care is a new concept in the Middle East, with varying levels of development across countries, Jordan and Saudi Arabia have made significant strides, established localized palliative care services, and integrated them into national health strategies. In Jordan, for example, the King Hussein Cancer Center has developed hospital-based palliative care teams and home care hospice services [6].

Understanding the factors contributing to readmissions and the impact of palliative care interventions is essential for improving patient outcomes, guiding policy decisions, and optimizing healthcare resource allocation.

### Objectives

The primary objective of this study is to describe the causes of hospital readmission from a hospice setting (home care and palliative clinic) within one week of discharge among adult cancer patients cared for at a comprehensive oncology center in Jordan. The secondary objective is to report the demographic and clinical characteristics of patients observed with these early readmissions.

## Methods

This study was approved by the Institutional Review Board (IRB) at King Hussein Cancer Centre (KHCC) in Amman, Jordan. KHCC is an internationally accredited, comprehensive cancer center that provides care for adult and pediatric patients with various malignancies from Jordan and the Middle East.

We conducted a retrospective descriptive study of palliative care patients who were readmitted to KHCC within one week of their most recent discharge from hospice settings (home care or clinic), over a 1-year period (January 2024 till December 2024). Data was obtained from the Computerized Patient Record System (CPRS), the electronic documentation system at KHCC. In this study, within one week of discharge refers to hospital readmission within seven days of the most recent discharge from an acute care hospitalization, after which patients had resumed hospice care in either the clinic or home setting. The 7-day window was chosen because early readmissions are more closely tied to modifiable discharge and transition-of-care factors and frequently more preventable than later readmissions [7].

Adult cancer patients ( > = 18 years old) who were followed by palliative care team and were admitted either to the inpatient ward or intensive care unit (ICU) from any hospice setting within one week of discharge during 2024 were included. At KHCC, hospice enrollment is documented through a structured process. When the primary oncologist determines that disease-directed anticancer therapy is no longer beneficial, this is discussed with the patient and/or family, and the patient is formally referred to the palliative care team for ongoing care until death. The palliative team conducts a family meeting to confirm understanding and agreement with goals of care. Following acceptance, the attending physician of record transitions from oncology to palliative care. For this study, hospice patients were those already under the palliative care service with documented hospice enrollment.

Hospice care is delivered by a multidisciplinary team of palliative care physicians, nurses, social workers, pharmacists, and spiritual counselors. Services include comprehensive symptom management, psychosocial support, medication provision (including opioids and adjuvant therapies), caregiver support, and 24‑hour access to clinical guidance. Care is provided at home through scheduled home‑care nursing visits or in clinic‑based hospice visits, depending on the Palliative Performance Scale (PPS) and patient needs, with inpatient hospice admissions when closer monitoring is required. The following data was extracted from each patient’s electronic records: Demographic variables ( age, gender, height, weight, Body Mass Index [8]), primary cancer diagnosis, comorbidities, code status (do-not-resuscitate or full code) [9], Palliative Performance Scale (PPS) [10], hospice setting before readmission (clinic versus home care), number of days between the two most recent admissions, renal functions (assessed using Creatinine Clearance by Cockcroft-Gault calculation and CKD classification) [11], liver function (assessed using Child Pugh score for severity of liver disease in adults) [12], hospital readmission causes, patient discharge place ( home, transferred to another hospital or died), and length of stay during the last admission. Physician and clinical pharmacist notes on the CPRS were used to verify the date, causes, and details of readmissions.

Physicians’ last discharge summary, clinical pharmacist discharge notes on the day of discharge, in addition to any note from the last discharge to current readmission (home care / clinic / emergency notes) used to assess the change of medications prior to readmission.

Readmission causes were classified into three categories based on recommendations from a palliative care provider [3]: uncontrolled conditions, unanticipated new medical issues, and a combined category of misunderstanding of hospice goals and caregiver distress. Patients could be classified into more than one category as multiple causes could contribute to a single readmission.

Uncontrolled symptoms were defined as worsening conditions related to the hospice diagnosis, such as increased edema in patients with congestive heart failure. Unanticipated new medical issues were defined as conditions not related to the hospice diagnosis (e.g., pneumonia, falls, or accidental disconnection of feeding tube). Disease progression was classified as an unanticipated new medical issue when it manifested as an acute, clinically destabilizing event that was not present or anticipated at the time of hospice discharge and required urgent inpatient management. Examples included hemorrhagic brain metastasis, vasogenic cerebral edema, acute worsening dyspnea and hepatic encephalopathy. These events represented sudden complications of advanced malignancy rather than gradual symptom worsening and were therefore analyzed separately from uncontrolled pre-existing hospice-related symptoms. Misunderstanding of hospice goals and caregiver distress were identified only when explicitly documented, such as prioritizing comfort over cure or misinterpreting symptom control measures-such as liberal use of morphine for pain- as hastening death. This was often associated with caregiver’s overestimation of the patient’s abilities and inadequate preparedness to manage increasing care demands as functional decline progressed [3].

In case the reason for readmission was not clearly documented, the chart was flagged for review by a second reviewer. Disagreements were resolved by consensus; if unresolved, a third reviewer served as an arbitrator. All reviewers had at least 2 years of experience in hospice and palliative care at KHCC.

Data was collected and managed using REDCap, a secure, web-based application for research data capture. REDCap was used to build Case Report Forms (CRFs) with built-in validation rules and branching logic to ensure data quality. Data entry was performed by trained personnel with restricted access based on role-specific permissions.

### Statistical analysis

Descriptive statistics were used to summarize the study data. Categorical variables, such as gender, hospice setting, code status, and reasons for readmission, were expressed as frequencies and percentages. Continuous variables, including age, BMI, time between admissions, and length of stay, were summarized using measures of central tendency (mean, median) and dispersion (standard deviation, range).

All readmission events were included in the analysis (*N* = 163). Baseline patient characteristics that do not change between admissions (e.g., age, sex, primary cancer, comorbidities) were analyzed once per patient (*N* = 133). Variables that may vary by admission (e.g., reason for readmission, code status at that admission, outcome) were analyzed at the event level. This approach avoids duplicating fixed characteristics while fully capturing each discrete readmission.

## Results

There were 163 readmission events among 133 patients; 24 patients experienced multiple readmissions. Patient‑level baseline characteristics are summarized for 133 unique patients (Table 1), and readmission‑level characteristics are summarized for 163 events (Table 2).

**Table 1:** Baseline characteristics (patient level, *n* = 133)

Variable	Level	Frequency (%)
Gender	Female	69 (51.9%)
	Male	64 (48.1%)
BMI	Normal weight	56 (42.1%)
	Obesity	32 (24.1%)
	Overweight	32 (24.1%)
	Underweight	13 (9.7%)
Any comorbidities	No	37 (27.8%)
	Yes	96 (72.2%)
Number of comorbidities for each patient	1	35 (36.4%)
	2	43 (44.8%)
	3	16 (16.7%)
	4	2 (2.1%)
Details of comorbidities	Cardiovascular	80 (83.3%)
	Endocrine	48 (50.0%)
	Mental health	15 (15.6%)
	Urogenital	14 (14.6%)
	Respiratory	11 (11.5%)
	Gastrointestinal	5 (5.2%)
	Musculoskeletal	2 (2.1%)
	Eye and ear	1 (1.0%)
	Skin	1 (1.0%)
Primary cancer site	Digestive Organs	37 (27.8%)
	Breast	26 (19.5%)
	Respiratory System & Intrathoracic Organ	17 (12.8%)
	Urinary Tract	8 (6.0%)
	Female Genital Organs	8 (6.0%)
	Connective, subcutaneous and other soft tissue	8 (6.0%)
	Eye, Brain, and other parts of CNS	7 (5.3%)
	Hematopoietic and Reticuloendothelial system	7 (5.3%)
	Lip, Oral Cavity and pharynx	5 (3.8%)
	Lymph-Nodes	3 (2.3%)
	Thyroid and Other Endocrine glands	3 (2.3%)
	Peripheral & Autonomic Nervous system	2 (1.5%)
	Retroperitoneum & Peritoneum	1 (0.8%)
	Male Genital Organs	1 (0.8%)

**Table 2:** Readmission characteristics (readmission level, *n* = 163)

Variable	Level	Frequency (%)
Hospice prior to readmission	Clinic	46 (28.2%)
	Home Care	117 (71.8%)
Code status	DNR	59 (36.2%)
	Full Code	104 (63.8%)
PPS	10	17 (10.4%)
	20	22 (13.5%)
	30	41 (25.2%)
	40	30 (18.4%)
	50	33 (20.2%)
	60	15 (9.2%)
	70	5 (3.1%)
Days between the two most recent hospital admissions	0	1 (0.6%)
	1	14 (8.5%)
	2	20 (12.3%)
	3	27 (16.6%)
	4	27 (16.6%)
	5	29 (17.8%)
	6	20 (12.3%)
	7	25 (15.3%)
Kidney function	Normal	96 (58.9%)
	AKI	41 (25.2%)
	AKI on CKD	17 (10.4%)
	CKD G1	5 (3.1%)
	CKD G3a	2 (1.2%)
	CKD G3b	2 (1.2%)
Liver function	Normal	103 (63.2%)
	Child-Pugh A	14 (8.6%)
	Child-Pugh B	18 (11.0%)
	Child-Pugh C	28 (17.2%)
Discharge status	Deceased	88 (54.0%)
	Discharged to home	75 (46.0%)

The median age of patients readmitted from a hospice setting was 59 years (range, 18–88 years). Of the 133 patients, 69 (51.9%) were female and 64 (48.1%) were male. Several patients had normal body weight (42.1%).

Most patients (*n* = 96, 72.2%) had at least one comorbidity, with many presenting multiple comorbid conditions. The most frequently reported comorbidities were cardiovascular (*n* = 80, 83.3%), endocrine (*n* = 48, 50.0%), mental health (*n* = 15, 15.6%), and urogenital (*n* = 14, 14.6%) disorders.

The most common primary diagnosis was gastrointestinal cancers (*n* = 37, 27.8%), followed by breast cancer (*n* = 26, 19.5%) and cancers of the respiratory system and intrathoracic organs (*n* = 17, 12.8%).

Most patients (*n* = 117, 71.8%) were receiving hospice care in the home setting prior to readmission and had a Full Code status (*n* = 104, 63.8%).

Regarding functional status, the median Palliative Performance Scale (PPS) score was 40 (range, 10–70). The median days between hospital readmission was 4 days (range, 0–7), and the median length of hospital stay during the last readmission was 5 days (range, 0–34).

Most patients had normal kidney (*n* = 96, 58.9%) and liver function (*n* = 103, 63.2%). At discharge, 88 patients (54.0%) had died during hospitalization, while the remainder were discharged home.

Hospital readmission causes are summarized in Table 3. Multiple causes could be recorded for a single readmission; subcategory percentages are calculated within each main cause.

**Table 3:** Readmission causes classifications (event level, *n* = 163)

Cause	Specify	Frequency (%)
Uncontrolled conditions		92 (56.4%)
	Worsening infection	20 (21.7%)
	Uncontrolled Pain	18 (19.6%)
	Hematologic disorder	13 (14.1%)
	Uncontrolled shortness of breath	12 (13.0%)
	Fluid overload	10 (10.9%)
	Electrolyte disturbances	9 (9.8%)
	Uncontrolled nausea and vomiting	6 (6.5%)
	Others	14 (15.2%)
Unanticipated new medical issues		86 (52.8%)
	New infection	36 (42.0%)
	Acute kidney injury	17 (19.8%)
	Disease progression	13 (15.1%)
	Electrolyte disturbances	11 (12.8%)
	Hematologic disorder	10 (11.6%)
	Device procedures (Lines insertion)	7 (8.1%)
	Cardiac problems	6 (7.0%)
	GI problems	6 (7.0%)
	Medication overdose	1 (1.2%)
	Pain	1 (1.2%)
Caregiver distress		20 (12.3%)

Uncontrolled conditions contributed to 92 readmissions (56.4%), followed by unanticipated new medical issues in 86 cases (52.8%).

Among uncontrolled conditions, the most frequently reported were worsening infection (*n* = 20, 21.7%), uncontrolled pain (*n* = 18, 19.6%), hematologic disorders (*n* = 13, 14.1%), uncontrolled shortness of breath (*n* = 12, 13.0%) and fluid overload (*n* = 10, 10.9%). Chest infections were the most common worsening infections (*n* = 7, 7.6%), followed by wound infections (*n* = 4, 4.3%). Among hematologic disorders, uncontrolled bleeding (*n* = 6, 6.5%) and chronic melena (*n* = 4, 4.3%) were most frequent. The most frequent cause of fluid overload was pleural effusion (*n* = 7, 7.6%).

Among unanticipated new medical issues, new infections (*n* = 36, 42.0%) and acute kidney injury (*n* = 17, 19.8%) were most frequent. Chest infections were the most common unanticipated new infection (*n* = 16, 18.6%), followed by sepsis (*n* = 12, 14%). Hypercalcemia was the most common electrolyte disturbance (*n* = 5, 5.8%), and the most frequently displaced line was the nephrostomy tube (*n* = 4, 4.7%). On the other hand, five patients (5.8%) were readmitted for end-of-life care.

## Discussion

Consistent with prior palliative care research, our results highlight that hospice patients are typically medically complex and fragile, often requiring urgent hospital-level interventions following discharge. This study adds to the limited body of literature on hospice readmissions by offering insights from Jordan, where palliative care services are still developing. By identifying patient characteristics and factors contributing to early readmissions, our results establish a foundation for strategies aimed at minimizing avoidable hospitalizations and aligning care more closely with patient–centered goals.

In our study, the median age of readmitted hospice patients was 59 years (range: 18–88). This is comparable to the cohort reported by Grim et al. (2010) [13], who observed a mean age of 61 years (range: 21–93) among unplanned readmissions in the United States. However, our patients were approximately 7 years younger than those described by Cao et al. (2020) [3], who reported a mean age of 66.7 years. The slightly younger median age in our sample may suggest differences in end-of-life priorities between older and younger patients [14]. Our study did not evaluate contributory factors. Regarding gender, our cohort demonstrated a slight female predominance (51.9%). This is comparable to Grim et al. (2010) [13], who reported an equal distribution between males and females (50% each). In contrast, Cao et al. (2020) [3] observed a male predominance (56.5%) in their cohort. Causal explanations are beyond the scope of our design.

In our study, 72.2% of readmitted hospice patients had at least one comorbid condition, with a considerable proportion presenting with multimorbidity. The most frequent comorbidities were cardiovascular diseases (83.3%), endocrine disorders (50.0%), mental health conditions (15.6%), and urogenital disorders (14.6%). This pattern mirrors international findings in palliative populations and highlights the complexity of managing these patients after discharge [16]. This high burden of comorbidities contributes to increased patient needs, which in turn may result in sub-optimal symptoms management, inability for caregivers to provide adequate care at home, and the requirement for a higher level of care. These findings highlight the importance of individualized discharge planning and enhanced home support tailored to patient’s multimorbidity profiles. Although our analysis did not directly examine these relationships, future research is needed to explore how factors such as age, gender and comorbidity profiles influence readmission risk, length of stay, and discharge outcomes in palliative care settings.

In our study, the most common primary diagnosis among readmitted hospice patients was gastrointestinal cancers (*n* = 37, 27.8%), followed by breast cancer (*n* = 26, 19.5%) and cancers of the respiratory system and intrathoracic organs (*n* = 17, 12.8%). This pattern reflects the high burden of gastrointestinal malignancies in our cohort and aligns with international findings. For example, studies from Taiwanese and German similarly identified respiratory and gastrointestinal malignancies as the leading causes of hospice readmissions [17, 18]. This global pattern underscores the symptom complexity associated with digestive-system cancers, their contribution to early hospital readmissions, and the significant demand for hospice care among this group.

Most patients (71.8%) were receiving hospice care in the home setting prior to readmission, and 63.8% had a Full Code status. Compared with Ankuda et al. (2018), where 12.9% of hospice enrollees elected Full Code status, our population is higher; our design does not allow inference about underlying reasons. Prior work highlights the importance of communication and anticipatory planning when Full Code Status is present in hospice [19, 20].

The median Palliative Performance Scale (PPS) score was 40 (range: 10–70), reflecting advanced functional impairment in this population. The median time to hospital readmission was 4 days (range: 0–7), with a median length of stay during the last readmission of 5 days (range: 0–34). Notably, more than half of the patients (*n* = 88, 54.0%) died during hospitalization, while the remainder were discharged home. These findings align with international evidence demonstrating that patients readmitted to hospice or palliative care settings often experience significant functional decline in limited survival. For instance, a study in a home-based hospice and palliative care program showed a median survival of 3, 5, 10, 19, 29.5, and 35 days for PPS scores of 10%, 20%, 30%, 40%, 50%, and 60%, respectively [21]. Additionally, a study in a Canadian palliative care program reported a median PPS score of 40% at the time of enrollment, with a median survival of 18 days [22]. The short interval between discharge and readmission in our population highlights the vulnerability of this population, while the high in-hospital mortality rate reflects the need for anticipatory palliative interventions, proactive care planning, and enhanced home support. Although PPS is a reliable survival predictor, our findings suggest that additional factors – including comorbidity burden, discharge setting, and code status - should also be considered in predicting outcomes. Future research should focus on improving prognostication in this population. Direct comparisons of hospice care provided in the home setting prior to readmission remain limited in international studies, warranting further investigation.

Uncontrolled conditions and unanticipated new medical issues are the leading causes of hospital readmissions among our population, accounting for 56.4% and 52.8% of cases, respectively. Among uncontrolled conditions, worsening infections, uncontrolled pain, and hematologic disorders were frequently observed. Common unanticipated new medical issues include new infections, acute kidney injury, electrolyte disturbances, and device related complications. Although disease progression is inherent to advanced cancer, certain manifestations may present abruptly and unpredictably, necessitating hospital readmission despite ongoing hospice care. Comparable results have been reported in prior investigations. Cao et al. (2020) reported that unanticipated new medical issues and uncontrolled symptoms were the most common reasons for hospital readmission, accounting for 44.6% and 33.8% of cases, respectively [3]. Grim et al. also found that the primary reason for readmission most often due to the “disease process” category (63%) [13]. Our results show that disease progression, sudden complications, and inadequately managed symptoms play a big role in unplanned hospital readmissions. This highlights the need for regular checks and timely intervention strategies in palliative care populations. It also shows how dynamic and unpredictable advanced illness can be, reinforcing the importance of close monitoring and timely outpatient interventions.

The frequent occurrence of infections—both worsening and newly emerging—highlights the challenges in managing infectious complications in this population. In addition, uncontrolled pain accounted for nearly one-fifth of all readmissions. This is consistent with prior studies reporting infections and poorly controlled pain as a common reason for unplanned readmissions [23]. These findings emphasize the critical need for effective infection control strategies and enhanced pain management protocols to reduce avoidable hospitalizations and improve the quality of end-of-life care; however, intervention effectiveness was not evaluated in this study.

Hematologic disorders, fluid overload, acute kidney injury, electrolyte disturbances, and device displacements are less frequently reported in palliative literature, making our findings a valuable contribution to understanding the broader spectrum of complications prompting readmission.

A small proportion of patients were readmitted primarily for end-of-life care. Preferences for place of care and death vary; an umbrella review indicates many prefer home while a minority prefer hospital, shaped by multiple factors. Our data were not designed to determine preference‑driven readmissions (24).

Caregiver-related issues (distress or misunderstanding of hospice status) also played an important role, accounting for 12.3% of readmissions in our findings. Cao et al. (2020) reports such factors, representing 21.6% of cases among patients discharged to home hospice. Differences versus international reports may relate to documentation practices and observation windows; our study cannot determine causation. Qualitative work suggests psychosocial factors may influence readmissions near end of life [3, 16]. [15]

### Limitations

This study has several limitations. First, it was conducted at a single comprehensive cancer center and used a retrospective descriptive design without inferential analyses or a comparison group; therefore, findings are not intended to imply causation or risk prediction. Second, while documentation in the electronic record was used to classify reasons for readmission and caregiver‑related factors, incomplete or variable documentation may have introduced misclassification. Third, although the study is single‑center, oncology patients at KHCC typically initiate and complete their care at this institution. Patients are not admitted to outside hospitals, and home‑based management is supervised by KHCC teams through clinic hospice or home‑care nursing, making missing external readmissions unlikely. Finally, broader social and system‑level factors (e.g., caregiver perspectives, community resources) were not captured.

## Conclusion

In this descriptive study, early hospital readmissions among hospice patients most often involved uncontrolled symptoms or new medical issues. Patients commonly had advanced functional impairment, short survival, and high in‑hospital mortality. These findings summarize patterns observed within our center and may support future efforts to better understand symptom management needs, transitions of care, and documentation practices in hospice populations.

## Data Availability

The corresponding author can provide the database created and used in the study upon reasonable request.

## Abbreviations

PPS: Palliative Performance Scale
KHCC: King Hussein Cancer Center
IRB: Institutional Review Board
DNR: Do-Not-Resuscitate
CPRS: Computerized Patient Record System
CKD: Chronic Kidney Disease
BMI: Body Mass Index
MRN: Medical Record Number
AKI: Acute Kidney Injury

## Declarations

### Corresponding author

Correspondence to Ala’a Dabbous.
